# SBP1 promotes tumorigenesis of thyroid cancer through TXN/NIS pathway

**DOI:** 10.1186/s10020-023-00700-y

**Published:** 2023-09-08

**Authors:** Jiancang Ma, Xin Huang, Jinkai Xu, Zongyu Li, Jingyue Lai, Yawei Shen, Jun Zhao, Xuejun Sun, Lieting Ma

**Affiliations:** 1 Department of General Surgery, The Second Affiliated Hospital of Xi’an Jiaotong University, Xi’an, Shaanxi 710004 China; 2 Department of General Surgery, Xi’an Central Hospital, Xi’an Jiaotong University, Xi’an, Shaanxi 710003 China; 3 Department of General Surgery, The First Affiliated Hospital of Xi’an Jiaotong University, No. 277 West Yanta Road, Xi’an, Shaanxi 710061 China; 4 Department of Laboratory Medicine, The First Affiliated Hospital of Xi’an Jiaotong University, No. 277 West Yanta Road, Xi’an, Shaanxi 710061 China

**Keywords:** Thyroid cancer, Selenium-binding protein1, Thioredoxin, Differentiation, Sodium/iodide symporter

## Abstract

**Background:**

As the tissue with the highest selenium content in the body, the occurrence and development of thyroid cancer are closely related to selenium and selenoproteins. Selenium-binding protein 1 (SBP1) has been repeatedly implicated in several cancers, but its role and molecular mechanisms in thyroid cancer remains largely undefined.

**Methods:**

The expression of SBP1, sodium/iodide symporter (NIS) and thioredoxin (TXN) were analyzed in clinical samples and cell lines. Cell counting kit-8 (CCK-8) and tube formation assays were used to analyze the cell viability and tube formation of cells. Immunofluorescence was used to determine the expression of the NIS. Co-immunoprecipitation (Co-IP) assay was carried out to verify the interaction of SBP1 with TXN. The mouse xenograft experiment was performed to investigate the growth of thyroid cancer cells with SBP1 knockdown in vivo.

**Results:**

SBP1 was significantly increased in human thyroid cancer tissues and cells, especially in anaplastic thyroid cancer. Overexpression of SBP1 promoted FTC-133 cell proliferation, and the culture supernatant of SBP1-overexpression FTC-133 cells promoted tube formation of human retinal microvascular endothelial cells. Knockdown of SBP1, however, inhibited cell proliferation and tube formation. Furthermore, overexpression of SBP1 inhibited cellular differentiation of differentiated thyroid cancer cell line FTC-133, as indicated by decreased expression of thyroid stimulating hormone receptors, thyroglobulin and NIS. Knockdown of SBP1, however, promoted differentiation of BHT101 cells, an anaplastic thyroid cancer cell line. Notably, TXN, a negative regulator of NIS, was found to be significantly upregulated in human thyroid cancer tissues, and it was positively regulated by SBP1. Co-IP assay implied a direct interaction of SBP1 with TXN. Additionally, TXN overexpression reversed the effect of SBP1 knockdown on BHT101 cell viability, tube formation and cell differentiation. An in vivo study found that knockdown of SBP1 promoted the expression of thyroid stimulating hormone receptors, thyroglobulin and NIS, as well as inhibited the growth and progression of thyroid cancer tumors.

**Conclusion:**

SBP1 promoted tumorigenesis and dedifferentiation of thyroid cancer through positively regulating TXN.

**Supplementary Information:**

The online version contains supplementary material available at 10.1186/s10020-023-00700-y.

## Background

The incidence of thyroid cancer has increased rapidly during the past few decades due to the growing use of diagnostic imaging and fine-needle aspiration biopsy, which has led to enhanced detection and diagnosis of subclinical thyroid cancers (Kitahara and Sosa 2016; Li et al. 2020). Generally, 90% of thyroid carcinomas are differentiated types that have an excellent prognosis and can be efficiently cured by conventional therapies (surgery followed by radio iodine therapy and suppression therapy with thyroid hormone); however, some patients can develop advanced disease such as poorly differentiated thyroid cancer (PDTC), metastatic differentiated thyroid cancer (DTC), and anaplastic thyroid cancer (ATC), that fails to respond to conventional therapies, resulting in high morbidity and mortality (Cabanillas et al. 2016; Hernando et al. 2020; Ibrahimpasic et al. 2019). Therefore, identifying the underlying mechanisms of ATC contributes to thyroid cancer therapy.

The thyroid gland has the highest selenium concentration of all tissues, twice as much as the kidney tissue with the second highest selenium content (Dickson and Tomlinson 1967). The occurrence and development of thyroid cancer are closely related to selenium and selenoproteins. Selenium is vital for many immune cell functions, ant it enhances T cell proliferation, natural killer cell activity, and immunity to pathogens (Avery and Hoffmann 2018). Meanwhile, high selenium exposure decreases the risk of specific types of cancer, including breast cancer, lung cancer, esophageal cancer, gastric cancer, and prostate cancer (Cai et al. 2016). However, randomized controlled trials showed that selenium supplementation even increased the risk of some types of cancer, such as advanced prostate cancer and skin cancer (Vinceti et al. 2017). Additionally, each tissue responds differently to selenium exposure, with brain selenium maintained at the expense of other tissues during selenium depletion (Burk and Hill 2009). These findings suggested that the study of selenium (and selenoproteins) and cancer is complicated by the existence of a diverse array of organic and inorganic selenium compounds. Selenium supplementation reduced serum thyroid peroxidase and thyroglobulin autoantibody levels in patients with chronic autoimmune thyroiditis (Wichman et al. 2016). The roles of selenium and selenoproteins need to be elucidated.

Selenium-binding protein 1 (SBP1, SELENBP1) belongs to the class of seleniumcontaining proteins that tightly bind selenium. The human SBP1 gene encodes a 472 amino acid protein that has a molecular weight equal to 56 kDa. SBP1 may play important roles in several fundamental physiological functions, including protein degradation, intra-Golgi transport, cell differentiation, cellular motility, redox modulation, and the metabolism of sulfur-containing molecules (Elhodaky and Diamond 2018; Porat et al. 2000). Yeast two-hybrid analysis confirmed that SBP1 interacts with von Hippel-Lindau protein (pVHL)-interacting deubiquitinating enzyme 1 (VDU1) and may play a role in ubiquitination/deubiquitination-mediated protein degradation pathways in a selenium-dependent manner (Jeong et al. 2009). Levels of SBP1 are expressed in a variety of cells and tissues (liver, heart, lung, and kidney) and have often been found to be decreased in human cancers as compared to the corresponding tissues. Lower levels are often associated with poor clinical outcome (Kim et al. 2006; Li et al. 2008; Xia et al. 2011; Zhang et al. 2011).

In this study, we observed that SBP1 is frequently upregulated in human thyroid cancer tissues and cells, especially in ATC. Functional studies show that SBP1 knockdown represses thyroid cancer cell growth and differentiation. Mechanistically, SBP1 promotes thyroid tumorigenesis through positively regulating thioredoxin (TXN).

## Materials and methods

### Tissue samples and cell culture

A thyroid tissue microarray (TH8010a, Alenabio, Xi’an, China) consisting of 70 patients, including 44 (62.8%) patients with papillary thyroid carcinoma (PTC), 20 (28.6%) patients with follicular thyroid carcinoma (FTC), and six (8.6%) patients with ATC and 10 thyroid normal tissue, was immunostained using anti-SBP1 antibody.

Twelve human FTC tissue samples and pair-matched adjacent non-neoplastic tissue samples were obtained from patients undergoing surgical resection at the Second Affiliated Hospital of Xi’an Jiaotong University. Informed consent was obtained from all patients. This study was carried out according to the Declaration of Helsinki and the Ethics Committee of Xi’an Jiaotong University approved the use of human tissue samples.

### Cell culture

The human normal thyroid cell Nthy-ori 3-1was purchased from Jennio biotech (Guangzhou, China). DTC cell line FTC-133 and human retinal microvascular endothelial cells (RCECs) were obtained from Procell life science and technology (Wuhan, China). DTC cell line B-CPAP, human BHT-101 cells and ACT cells were obtained from Type Culture Collection of the Chinese Academy of Sciences (Shanghai, China). The Nthy-ori 3 − 1, BHT101, B-CPAP, BHT-101 and ACT cell line were maintained in RPMI 1640 medium (Invitrogen, Carlsbad, CA, USA) supplemented with 10% fetal bovine serum (FBS) (Sigma, St. Louis, MO, USA), 100 U/ml of penicillin G and 100 μg/ml of streptomycin. RCECs were cultured in Dulbecco’s Modified Eagle’s medium (DMEM) (Gibco Life Technologies, Rockville, MD, USA) supplemented with 10% FBS, 100 U/ml of penicillin G and 100 μg/ml of streptomycin. The cultures were maintained in a humidified atmosphere of 5% CO_2_ and 95% air at 37 ˚C.

### Immunohistochemistry

For immunohistochemistry (IHC), four TFC, four PTC and four ATC thyroid cancer tissues as well as their pair-matched adjacent non-neoplastic tissue were fixed in 10% formalin and embedded in paraffin. Slides were deparaffinized and serially rehydrated in accordance with standard procedures. After being incubated in 0.3% H_2_O_2_ in Tris Buffered Saline for 15 min, the slides were treated with 0.01 M citrate buffer (pH = 6.0) at 120 °C for antigen retrieval, blocked in 10% normal serum, and then immunostained with anti-SBP1 antibody (5 μg/ml, #ab90135, Abcam, Cambridge, UK) or anti thioredoxin antibody (1:200, # ab185329, Abcam)) overnight at 4 °C. After that, biotin-labeled anti-mouse secondary antibody was added to the slides. Then streptavidin-peroxidase was applied. DAB and hematoxylin were used to detect the immunoreactivity of the primary antibody. Slides were scanned at 20× magnification using an Olympus BX53 (Olympus, Osaka, Japan). Each sample was analyzed three times, and a representative figure of SBP1 expression in thyroid cancer tissue and adjacent non-neoplastic tissue was presented.

### Immunofluorescence

For immunofluorescence (IF), cells were fixed with 4% paraformaldehyde for 15 min. After being blocked with 5% BSA for 1 h, the cells were immunostained with SLC5A5 Polyclonal Antibody (1:20, ThermoFisher scientific, Waltham, MA, USA) overnight at 4 °C. After that, cells were incubated with Alexa Fluor ® 594 secondary antibody (1:400) for 1 h, and then incubated with 1 ug/ml Hoechst for 10 min. After a mount coverslip, the slides were scanned at 20× magnification using an Olympus FV3000 microscope (Olympus, Osaka, Japan).

### Cell transfection

BHT101 cells were plated in a 6-well culture plate at a density of 2 × 10^6^ cells/well. BHT101 cells were transfected with 50 pmol of SBP1 small interfering RNA (siRNA) using Roche’s XtremeGene siRNA transfection reagent (Roche, Basel, Switzerland) according to the manufacturer’s instructions. The experimental groups were as follows: Control group (BHT 101 cells without any treatment), si-NC group (BHT 101 cells were transfected with si-NC as a negative control), and si-SBP1 group (BHT 101 cells were transfected with SBP1 siRNA).

For SBP1 knockdown and TXN overexpression, 25 pmol of SBP1 siRNA and 25 pmol of TXN overexpression plasmid were transfected to BHT101. The ratio of transfection reagent to plasmid DNA was 3:1. The experimental groups were as follows: si-SBP1 + NC group (BHT 101 cells were co-transfected with SBP1 siRNA and negative control plasmid), si-SBP1 + TXN group (BHT 101 cells were co-transfected with SBP1 siRNA and TXN overexpression plasmid).

FTC-133 cells were plated in a 6-well culture plate at a density of 2 × 10^6^ cells/well. FTC-133 cells were transfected with SBP1 overexpression lentiviral vector (MOI = 5, OBIO technology) using Roche’s XtremeGene HP DNA transfection reagent (Roche) according to the manufacturer’s instructions. The experimental groups were as follows: Control group (FTC-133 cells without any treatment), NC group (FTC-133 cells were transfected with empty lentiviral vector), and SBP1 group (FTC-133 cells were transfected with SBP1 overexpression lentiviral vector).

### Real-time quantitative PCR

Total RNA was extracted from BHT101 or FTC-133 cells using TRIzol reagent (Invitrogen). To obtain cDNA, 100 ng of extracted RNA was reverse-transcribed using the RevertAid First Strand cDNA Synthesis Kit (Invitrogen). Real-time quantitative PCR (RT-qPCR) analyses were performed by using PrimeScript RT-PCR kits (Takara, Dalian, China). The mRNA level of β-actin was used as an internal control. All expressions were calculated using the 2^−ΔΔCt^ method (Livak and Schmittgen 2001). The primers for RT-qPCR are as follows: TTF1 forward: 5’- ACGTGAGCAAGAACATGG − 3’, TTF1 reverse: 5’- CAGGTACTTCTGTTGCTTGA − 3’; TTF-2 forward: 5’- CCACAACCTCACACTCAA − 3’, TTF2 reverse: 5’- GCTCTCGAACATGTCCTC − 3’; TSH-R forward: 5’- CAACCCGTGTGAAGACATAA − 3’,TSH-R reverse: 5’- TGGCTGGTGAGGAGAATAA − 3’; TG forward: 5’- GGAGGGAGACAAGAAGATTTG − 3’, TG reverse: 5’- GCCCACAGTTGGCTTATT − 3’; NIS forward: 5’- GTACACCGGCATCGTAATC − 3’, NIS reverse: 5’- CGTGTAGAAGGTGCAGATAAT − 3’; SBP1 forward: 5’- GTCTACCTGCCCTGCATTTA − 3’, SBP1 reverse: 5’- GTGGATGACCTGGCAATACT − 3’; TXN forward: 5’- GTGGATGACTGTCAGGATGTT − 3’, TXN reverse: 5’- GACTAATTCATTAATGGTGGCTTCA − 3’; β-actin forward: 5’- TGACGTGGACATCCGCAAAG − 3’, β-actin reverse: 5’- TCTTCATTGTGCTGGGTGCC − 3’.

### Western blot analysis

Total protein was extracted from thyroid cancer tissue or thyroid cancer cells using RIPA buffer (Sigma-Aldrich, St. Louis, MO, USA) with a supplementary protease inhibitor cocktail (Roche). Then, the protein concentration was measured by the BCA protein assay kit (Pierce, Rockford, IL, USA) and 25 μg of lysates were electrophoresed on 10% SDS-PAGE and transferred to a nitrocellulose membrane (Bio-Rad Laboratories, Hercules, CA, USA). After blocking with 5% BSA, the membranes were incubated overnight with the indicated primary antibody at 4 °C, followed by incubated with Goat anti-Rabbit IgG (H + L) Secondary Antibody, HRP conjugate (1:10000, #31,460, ThermoFisher scientific) or Goat anti-Mouse IgG (H + L) Secondary Antibody, HRP conjugate (1:5000, #31,430, ThermoFisher scientific) for 1 h at 37 °C. Protein bands were visualized using the Enhanced Chemiluminescence System (Amersham Pharmacia Biotech, Buckinghamshire, United Kingdom). The primary antibodies used for the Western blot were as follows: anti-SBP1 antibody (1 μg/ml, #ab90135, Abcam), TSH-R polyclonal Antibody (0.5 μg/ml, # PA5-95449, ThermoFisher scientific), Thyroglobulin polyclonal antibody (1:2000, #PA5-102938, ThermoFisher scientific), NIS (SLC5A5) Polyclonal Antibody (1:1000, #PA5-97231, ThermoFisher scientific) and anti thioredoxin antibody (1:2000, # ab185329, Abcam). To detect protein expression in human tissues (n = 12) and animal tissues (n = 6), protein expression in each sample was measured three times. To detect protein expression in cell lines, three independent experiments, each repeated in triplicate was conducted.

### Cell proliferation

A cell counting kit-8 (CCK-8) assay was performed to analyze cell proliferation using CCK-8 detection kits (7sea biotech, Shanghai, China). Briefly, a 5 × 10^3^/well of cells were seeded onto a 96-well plate and cultured for 24 h. Then, the cells were rinsed and incubated with 10 μl of CCK-8 at 37 °C for 1 h. The absorbance values at 450 nm were analyzed. The experimental groups are as follows: Control group in FTC-133 cells, NC group, and SBP1 group; as well as Control group in BHT 101 cells, si-NC group, si-SBP1 group, si-SBP1 + NC group, and si-SBP1 + TXN group. Each group has three samples, each sample was tested three times.

### Tube formation assay

RCECs were suspended with a culture supernatant of SBP1 siRNA transfected BHT101 cells or SBP1 overexpression transfected FTC-133 cells. Then, 150 μl of RCECs with a density of 5 × 10^5^ cells/well were plated on a 96-well plate coated with Matrigel™. After 6 h, tube formation was counted with a microscope. The culture supernatants of BHT101 cells or FTC-133 cells treated with 10ng/ml of VEGF was used as positive control.

### GEPIA analysis

The expressions of TXN and the correlation between TXN and clinical stage in thyroid cancer patients were analyzed using GEPIA2 (http://gepia2.cancer-pku.cn/#index) (Tang et al. 2017). Based on the Cancer Genome Atlas (TCGA) and the Genotype-Tissue Expression (GTEx) projects, GEPIA2 is an interactive web for processing high-throughput RNA sequencing data of tumorous and normal samples. The present analysis included 512 thyroid carcinoma samples and 337 normal thyroid tissue samples. The “Box Plot” in the expression DIY module was used for analyzing the expression level of TXN in thyroid carcinoma samples and normal thyroid tissue samples. *P* value was set to 0.01, and the cut-off value of log_2_FC was set to 1. The “Stage Plot” in the expression DIY module was used to generate TXN expression violin plots based on the pathological stage of thyroid cancer patients. Major stage was used for plotting and the log_2_(TPM + 1) was used for Log scale.

### Co-immunoprecipitation (Co-IP) assays

After achieve 90% confluence, BTH101 cells were lysed in 20 mM Tris-HCl, pH 8.0, 150 mM NaCl, 1 mM EDTA, and 0.5% Nonidet P-40. Then the protein concentration was quantified using a BCA protein assay kit. Equal mass of protein was immunoprecipitated with anti-SBP1 antibody (5 μg/ml, #ab90135, Abcam). The immunocomplex was captured with protein G-agarose beads at 4 °C for 8 h, and normal mouse IgG (Santa Cruz Biotechnology) was applied as negative control. Beads were quickly washed three times with 100 mM KCl, 0.05% NP40. The samples were applied to SDS-PAGE and then immunoblotted with antibody against SBP1 (1 μg/ml, #ab90135, Abcam) or TXN (1:1000, # ab185329, Abcam).

### Animal experiments

We bought 4-week-old female BALB/c nude mice from Animal Experiment Center of Xi’an Jiaotong University (Xi’an, China) and quarantined them for 1 week before the experiments. BHT101 cells were infected with indicated shRNAs. After 72 h puromycin selection, cells were harvested and subcutaneously injected into nude mice for xenografts assay. We suspended the cells (3 × 10^6^) by 100 μl of PBS, then subcutaneously injected into the BALB/c mice. We measured the weight of the mice and the volume of the tumors every 2 days for nearly 3 weeks. The mice were killed at the end of the experiments, and then we excised the tumors, photographed them, and processed them for Western blot analyses. The experimental groups were as follows: Control group (nude mice without any treatment), sh-NC group (nude mice were subcutaneously injected with sh-NC infected-BHT101 cells) and sh-SBP1 group (nude mice were subcutaneously injected with sh-SBP1 infected-BHT101 cells). Each group included six nude mice. All animal experiment procedures were approved by the Animal Care and Use Committee of Xi’an Jiaotong University, and every effort was made to reduce the suffering of animals. The animal experiments strictly followed the ethical guidelines.

### Statistical analyses

All data are presented as the mean ± standard deviation. Differences between two groups were compared using unpaired *t* tests. Differences between multiple groups were compared using one-way analysis of variance (ANOVA) followed by the Bonferroni test. Values were considered statistically significant at *P* < 0.05. All the samples were run individually and averaged.

## Results

### SBP1 was upregulated in thyroid cancer

**Fig. 1:**
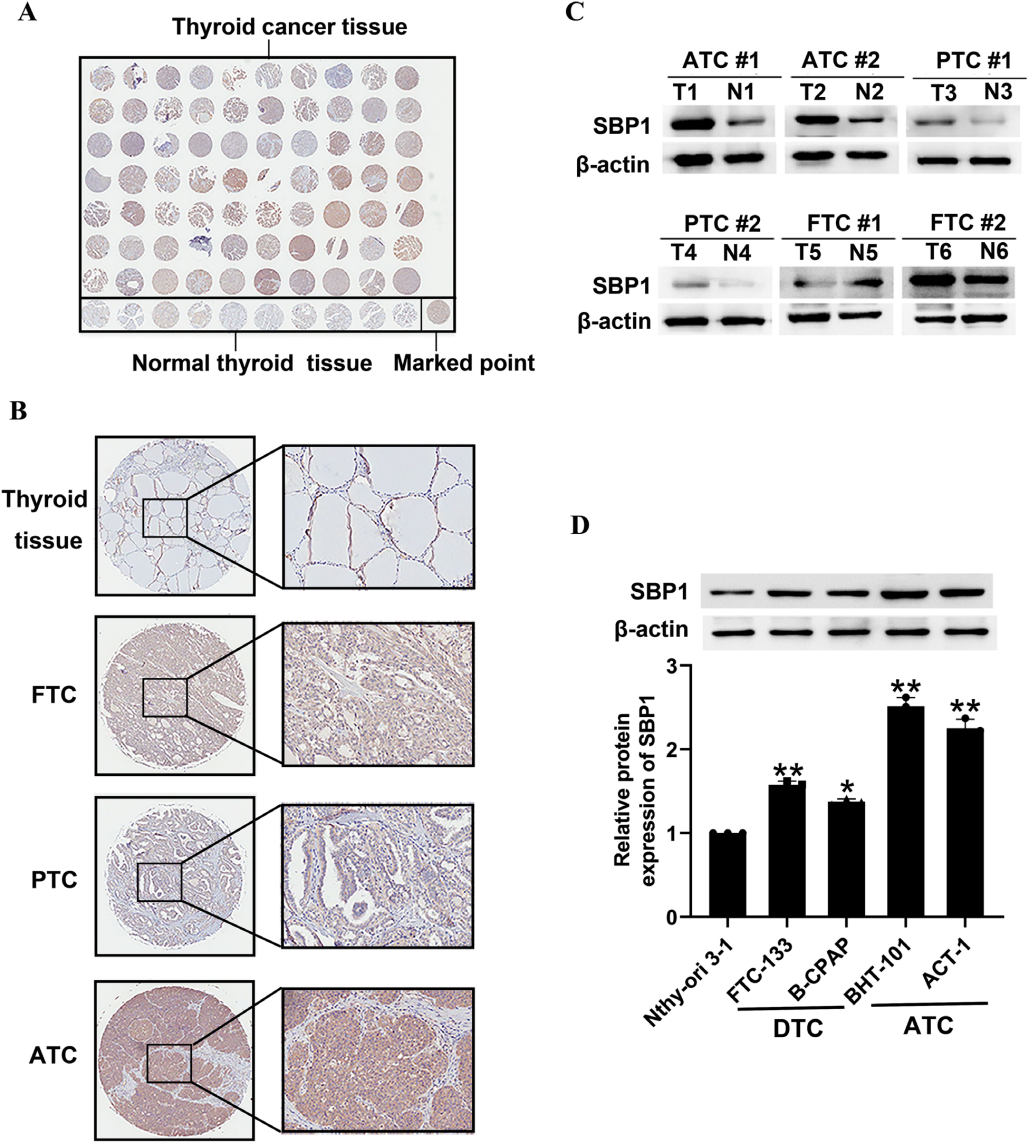
SBP1 was upregulated in thyroid cancer. (**A**) Immunohistochemical (IHC) staining of the expression of SBP1 in a human thyroid tissue microarray. (**B**) IHC staining of SBP1 in representative thyroid cancer tissue and normal thyroid tissues (magnification, left, 5×; right, 20 ×). (**C**) SBP1 expression was determined by Western blot analysis in 12 pairs of primary thyroid cancer and their matched noncancerous thyroid tissues. (**D**) Western blot analysis of SBP1 level in human normal thyroid cells, DTC cells, and ATC cells. DTC, differentiated thyroid cancer; ATC, anaplastic thyroid cancer; FTC, follicular thyroid cancer; PTC, papillary thyroid cancer. ^*^P < 0.05, ^**^P < 0.01 vs. Nthy-ori 3 − 1

A thyroid tissue microarray, consisting of 70 patients, including 44 (62.8%) papillary patients with thyroid carcinoma (PTC), 20 (28.6%) patients with follicular thyroid carcinoma (FTC), 6 (8.6%) patients with ATC and 10 patients with normal thyroid tissue, were collected. We observed frequent SBP1 upregulation in thyroid cancer samples compared to adjacent normal tissue (Fig. 1A). As shown in Fig. 1B, thyroid cancer tissues, especially ATC tissues, exhibited relatively strong staining for SBP1, and showed clear SBP1 plasma staining in ATC tissues. However, thyroid normal tissues showed clear SBP1 nuclear staining. Next, we analyzed the expression of SBP1 in twelve tissue samples of human FTC and adjacent pair-matched non-neoplastic tissue samples. Western blot data clearly showed a progressive upregulation of SBP1 (Fig. 1C). Compared to normal thyroid cells, SBP1 was significantly increased in human thyroid cancer cells, especially in ATC cell lines BHT-101 cells and ACT-1 cells (Fig. 1D). These results suggested that SBP1 may possess an unknown function in the control of thyroid cancer cell growth and progression.

### SBP1 promotes cell growth and tube formation of thyroid cancer cell lines

**Fig. 2:**
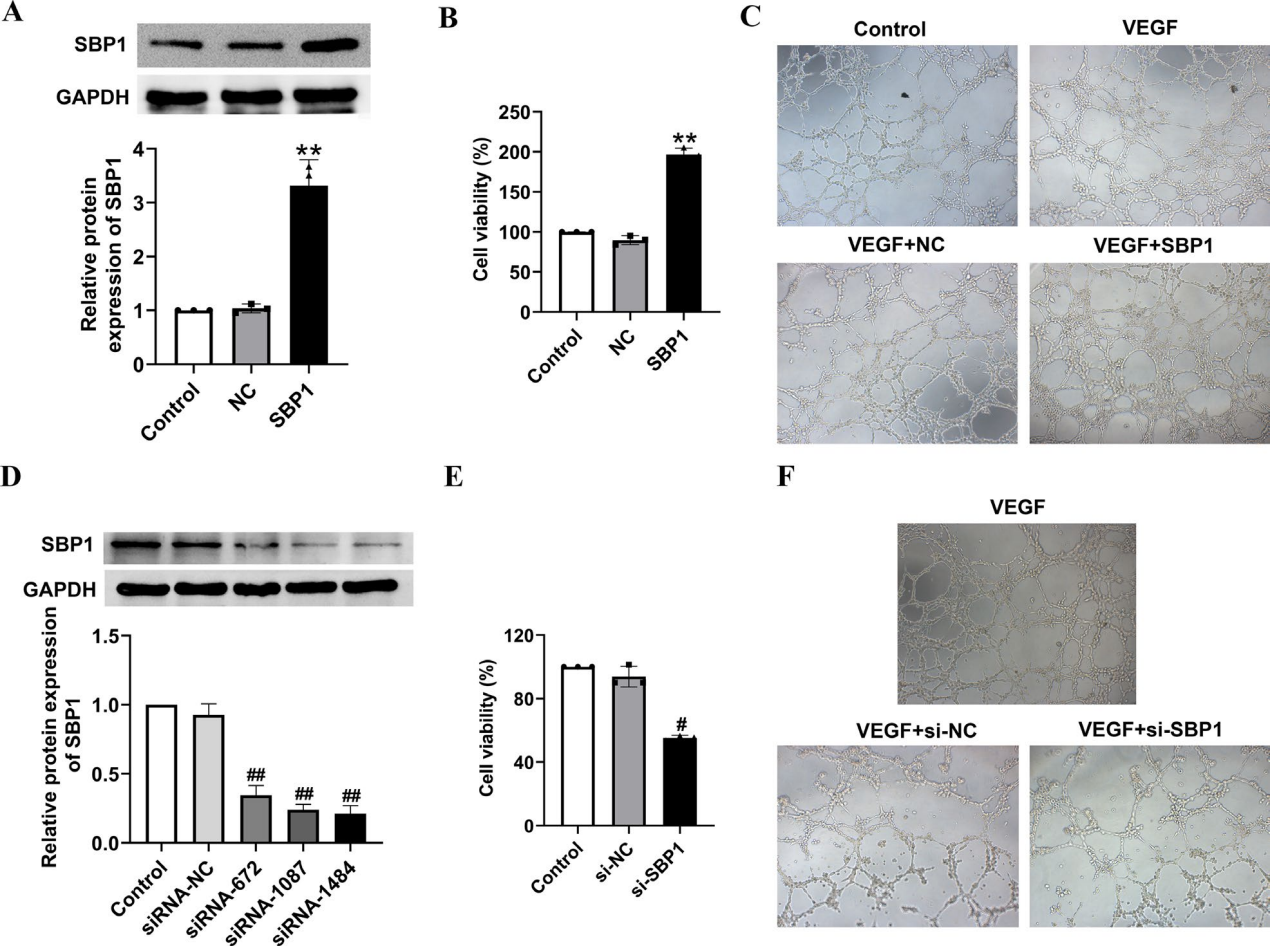
SBP1 promotes cell growth and tube formation of thyroid cancer cell lines. FTC133 cells were transfected with the SBP1 overexpression vector, while BHT101 cells were transfected with the SBP1 siRNA. (**A**) Western blot assays were performed to assess transfection efficiency of SBP1 overexpression in FTC133 cells. (**B**) FTC133 cell viability was analyzed by CCK-8 assay. (**C**) FTC133 cells were transfected with SBP1 overexpression vector and the culture supernatant was cultured with human retinal microvascular endothelial cells (RCECs). Then tube formation was analyzed by tube formation assay. (**D**) Western blot assays were performed to assess transfection efficiency of SBP1 knockdown in BHT101 cells. (**E**) Cell viability was analyzed by CCK-8 assay. (**F**) BHT101 cells were transfected with SBP1 siRNA and the culture supernatant was cultured with RCECs, then tube formation was analyzed by tube formation assay. **P < 0.01, vs. negative control (NC), ^#^*P* < 0.05, ^# #^*P* < 0.01, vs. siRNA negative control (si-NC).

To test the biological relevance of SBP1 expression in thyroid cancer cells, we examined the effect of SBP1 overexpression and knockdown on cellular functions. Overexpression of SBP1 was tested in FTC-133 cells as it is a differentiated thyroid cancer cell line which is commonly used. Western blot assay showed a significant higher expression of SBP1 after SBP1 overexpression (Fig. 2A). Overexpression of SBP1 induced an approximately 2 fold increase in cell viability when compared to the negative control vector (Fig. 2B). Additionally, tube formation was increased in SBP1 overexpression FTC133 cells (Fig. 2C). SBP1 knockdown was performed in BHT101 cells as it has the highest expression of SBP1 and it is a thyroid anaplastic carcinoma cell line derived from an undifferentiated primary carcinoma of the thyroid. SBP1 knockdown was performed by transfecting BHT101 cells with siRNA against SBP1, and si-SBP1 (#1484) was used for further study as it downregulated the most of SBP1 expression (Fig. 2D). Knockdown of SBP1 showed both greatly reduced cell viability (Fig. 2E) and tube formation compared to cells harboring control siRNA (Fig. 2F).

### SBP1 promotes dedifferentiate of thyroid cancer cell lines

**Fig. 3:**
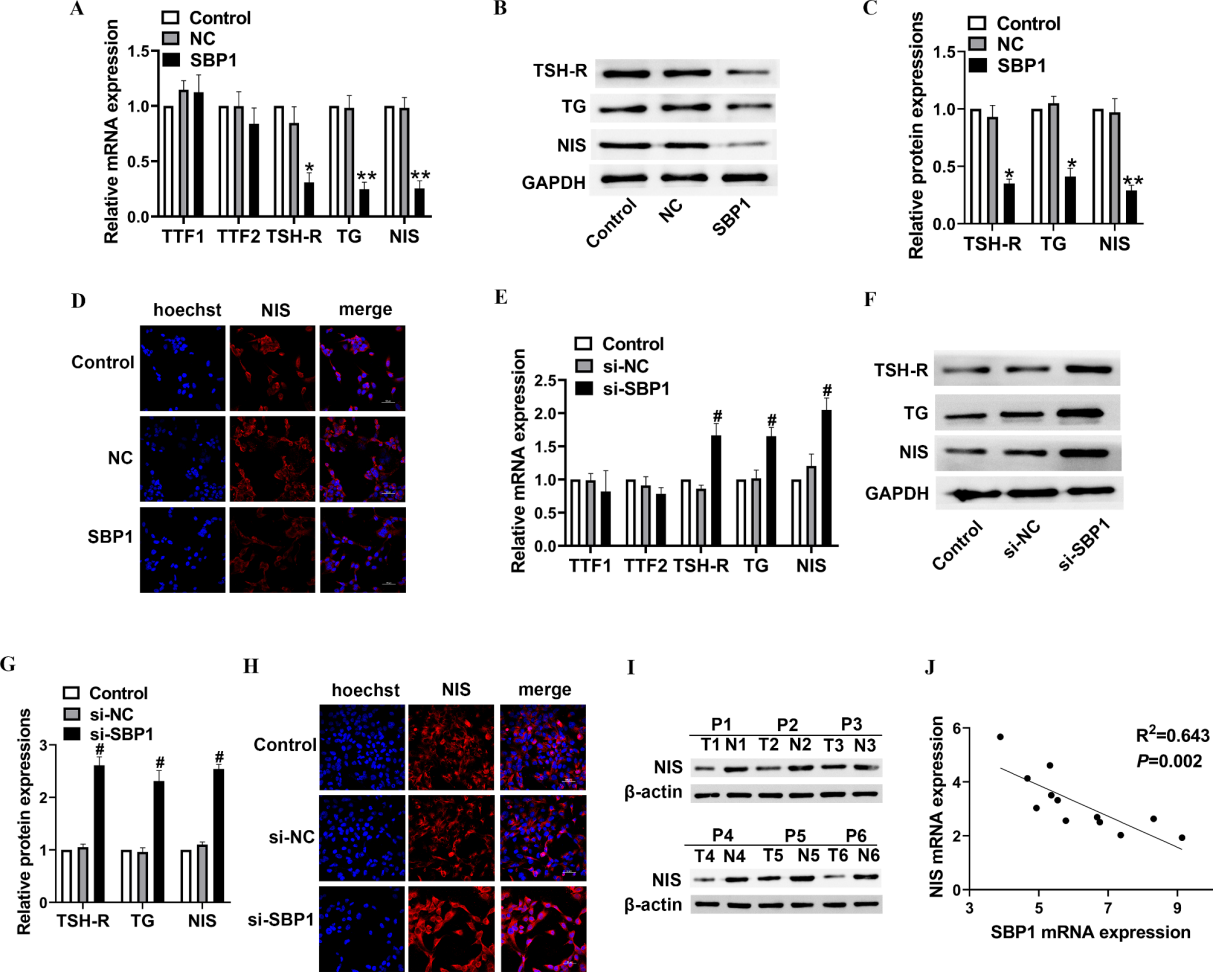
SBP1 promotes dedifferentiation of thyroid cancer cell lines. FTC133 cells were transfected with SBP1 overexpression vector, while BHT101 cells were transfected with SBP1 siRNA. (**A**) qRT-PCR assay were performed to assess mRNA levels of thyroid transcription factor-1 (TTF-1), thyroid transcription factor-1 (TTF-2), thyroid stimulating hormone receptor (TSH-R), thyroglobulin (TG) and sodium/iodide symporter (NIS) in FTC133 cells. (**B**) Western blot assays were performed to assess protein expression of TSH-R, TG and NIS in FTC133 cells. (**C**) Quantity analysis of protein expression of TSH-R, TG and NIS in FTC133 cells. (**D**) Immunocytochemical staining of NIS in FTC133 cells transfected with SBP1 overexpression vector. Scale bars: 50 μm. (**E**) qRT-PCR assay were performed to assess mRNA levels of TTF-1, TTF-2, TSH-R, TG and NIS in BHT101 cells. (**F**) Western blot assays were performed to assess protein expression of TSH-R, TG and NIS in BHT101 cells. (**G**) Quantity analysis of protein expressions of TSH-R, TG and NIS in BHT101 cells. (**H**) Immunocytochemical staining of NIS in BHT101 cells transfected with SBP1 overexpression vector. Scale bars: 50 μm. (**I**) NIS expression was determined by Western blot analysis in 12 pairs of primary thyroid cancer and their matched noncancerous thyroid tissues. (**J**) SBP1 was negatively correlated with NIS expression in 12 thyroid cancer tissues. *P < 0.05, **P < 0.01, vs. negative control (NC), ^#^*P* < 0.05 vs. siRNA negative control (si-NC).

Next, we evaluated the expressions of thyroid transcription factor (TTF)-1 and − 2 as well as the expression of differentiation related genes, such as thyroid stimulating hormone receptor (TSH-R), thyroglobulin (TG) and sodium/iodide symporter (NIS) in thyroid cancer cell lines. We identified a significant downregulation of differentiate related genes TSH-R, TG and NIS expression in SBP1 overexpression FTC-133 cells, whereas other thyroid transcription factor TTF-1 and TTF-2 revealed no significant differences after SBP1 overexpression (Fig. 3A-C), indicating an inhibited differentiation of FTC-133 cells after SBP1 overexpression. What is more, confocal analyses revealed a downregulation of NIS after SBP1 overexpression (Fig. 3D).

Additionally, the expression of TSH-R, TG and NIS in BHT101cells transfected with SBP siRNA were increased significantly compared to cells receiving control siRNA treatment in BHT101 cells (Fig. 3E-G). As shown in Fig. 3H, knockdown of SBP1 consistently increased NIS levels in BHT101 cells. These results imply that SBP1 promotes cell dedifferentiate of thyroid cancer cell lines and NIS plays a major role in it. Additionally, the expression of NIS was significantly decreased in human thyroid cancer tissues compared with that in pair-matched adjacent non-neoplastic tissues (Fig. 3I). A negative correlation was found between SBP1 and NIS mRNA expression (R^2^ = 0.643, *P* = 0.002, Fig. 3J).

### SBP1 binds directly to TXN

**Fig. 4:**
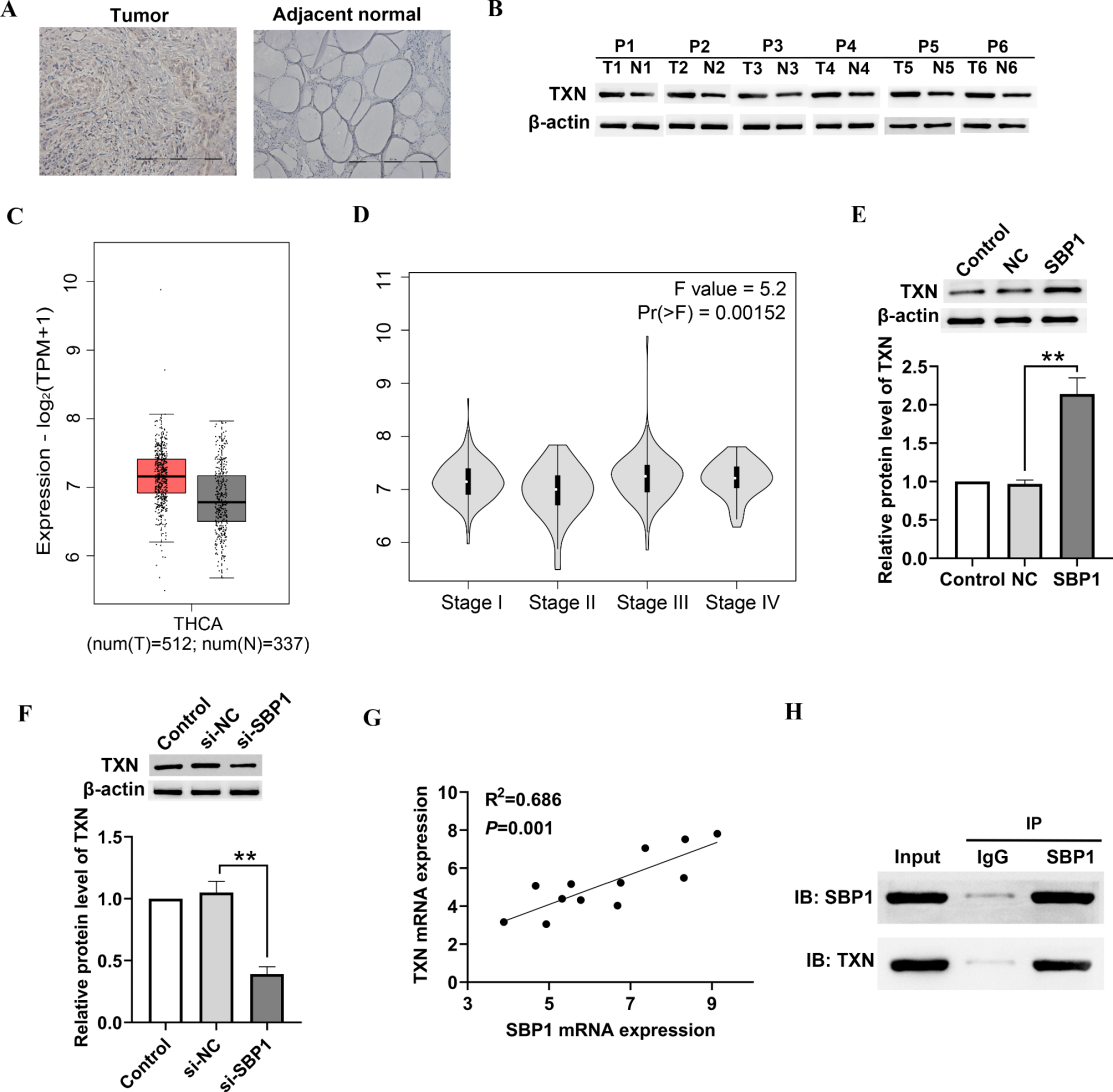
SBP1 interacts with TXN. (**A**) Immunohistochemistry staining of TXN in primary thyroid cancer and matched noncancerous thyroid tissue. (**B**) TXN expression was determined by Western blot analysis in 6 pairs of primary thyroid cancer and their matched noncancerous thyroid tissues. (**C**) The level of TXN in thyroid patients was analyzed. (**D**) Expression of TXN was positively correlated with thyroid cancer stage. (**E**) Western blot assays were performed to assess protein expression of TXN in BHT101 cells transfected with si-SBP1. (**F**) Western blot assays were performed to assess protein expression of TXN in FTC-133 cells transfected with SBP1 overexpression vector. (**G**) SBP1 was positively correlated with TXN expression in 12 thyroid cancer tissues. (**H**) Interaction of SBP1 and TXN in BHT101 cells was analyzed by co-immunopercipient. *P < 0.05. T, tumor; N, normal; si-NC, negative control siRNA; NC, negative control

**Fig. 5:**
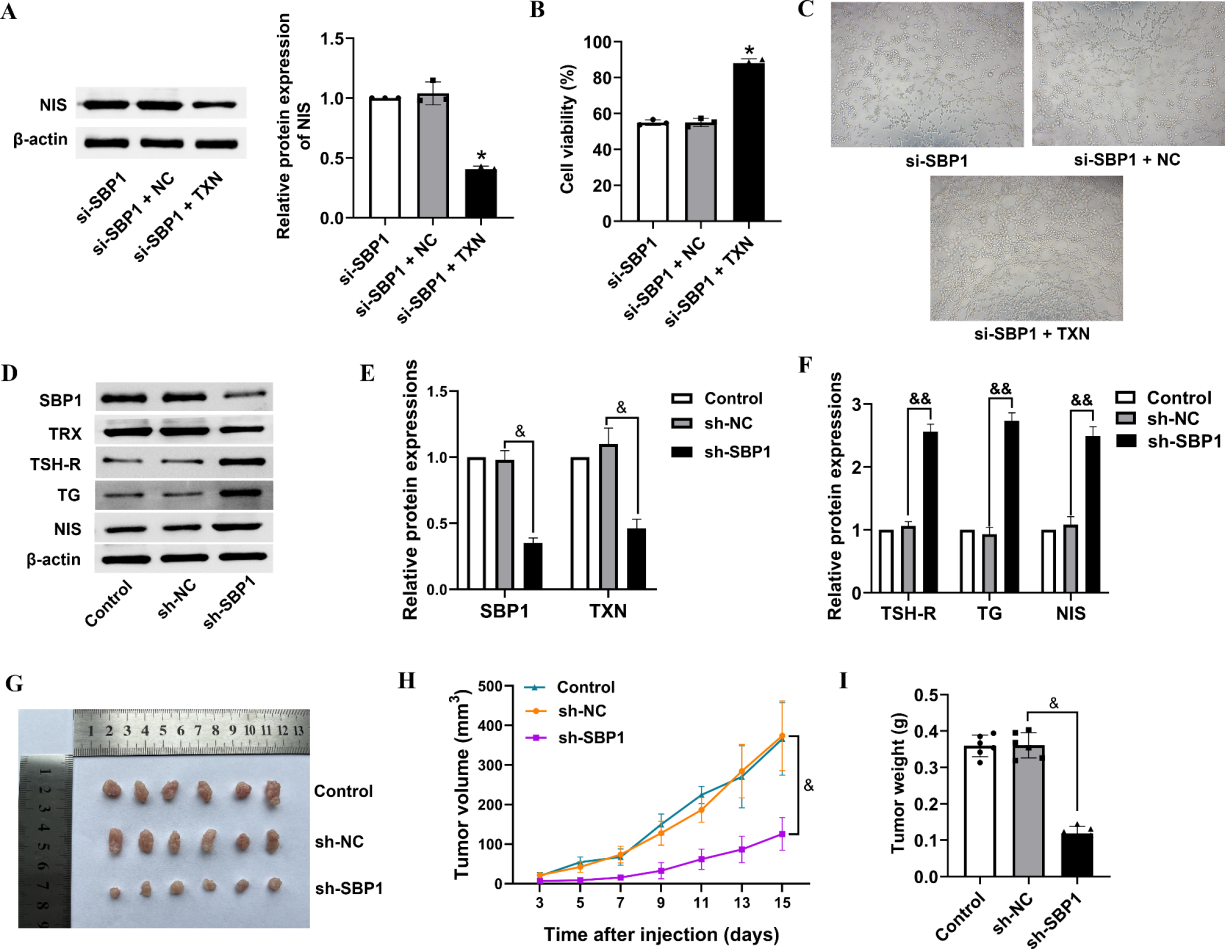
Knockdown of SBP1 inhibits xenograft tumor growth. BHT101 cells were transfected with si-SBP1 with or without TXN overexpression plasmid. (**A**) Western blot assays were performed to assess protein expression of NIS. (**B**) The cell viability was determined by CCK-8 assay. (**C**) Tube formation was analyzed by tube formation assay. (**D**) Tumor tissues from sh-SBP1 and sh-NC mice were homogenate and lysed. The protein levels of SBP1, TXN, TSH-R, TG, and NIS were analyzed by Western blot. (**E**) Quantity analyze of protein expressions of SBP1 and TXN. (**F**) Quantity analyze of protein expressions of TSH-**R**, TG, and NIS. (**G**) Photographs of dissected tumors from nude mice. (**H**) Tumor growth curves were compared between thyroid cells transfected by sh-SBP1 and sh-NC cells in nude mice. (**I**) Histogram represents mean tumor weight of dissected tumors from SBP1-knockdown and sh-NC mice, respectively. *P < 0.05 vs. si-SBP1 + NC; ^&^*P* < 0.05, ^&&^*P* < 0.01

It is reported that NIS expression depends on thioredoxin/thioredoxin reductase-1 (Txn/TxnRd1) mediated redox reactions (Leoni et al. 2016). To gain insight into the TXN expression that might contribute to the progress of thyroid cancer, TXN expression in human thyroid tumors was analyzed. We observed that TXN expression levels were significantly higher in thyroid cancer than in benign thyroid samples (Fig. 4A). Western blot assay demonstrated that the expression of TXN was highly increased in thyroid cancer samples compares to normal samples (Fig. 4B). By searching the bioinformatics website GEPIA2, we observed that the level of TXN was increased in thyroid cancer patients than in normal control (Fig. 4 C). In addition, TXN expression is positively correlated with the thyroid cancer stage (Fig. 4D). What is more, the protein expression of TXN in SBP1 knockdown BHT101 cells, as well as in SBP1 overexpression FTC-133 cells was analyzed. Overexpression of SBP1 increased the expression level of TXN in FTC-133 cells (Fig. 4E), while a significant reduction of TXN in si-SBP1 transfected BHT101 cells was found (Fig. 4F). TXN mRNA expression was positively correlated with the SBP1 mRNA expression (R^2^ = 0.686, *P* = 0.001, Fig. 4G).To explore whether SBP1 directly binds with TXN, we performed co-immunoprecipitation of endogenous of endogenous SBP1 in BHT101 cells. Western blot assay of immunoprecipitated SBP1 revealed that it strongly binds with TXN (Fig. 4H). Besides that, overexpression of TXN reversed the effect of SBP1 on cell growth and differentiation, as indicated by a decrease of NIS (Fig. 5A), an increase of cell growth and tube formation (Fig. 5B-C) in BHT101 cells co-transfected with TXN overexpression and SBP1 knockdown vector.

### Knockdown of SBP1 inhibits xenograft tumor growth

Thyroid cancer xenograft model in nude mice was established to investigate the role of SBP1 in vivo. We injected nude mice with BHT101 cells stably expressing SBP1-shRNAs or the control shRNAs. The expression of SBP1, TXN, TSH-R, TG and NIS in tumor tissues was analyzed by Western blot (Fig. 5D). Knockdown of SBP1 significantly down-regulated SBP1 and TXN expressions in thyroid tissue of nude mice (Fig. 5E). What is more, the expressions of TSH-R, TG and NIS were increased after SBP1 knockdown, indicating a transition from undifferentiated state to differentiated state (Fig. 5F). Tumors in the SBP1 knockdown mice grew slower than those in the mice with the control shRNA (Fig. 5G-H). Besides that, the tumor weight was significantly decreased in sh-SBP1 transfected nude mice (Fig. 5I).

## Discussion

In the present study, we have identified a high level of SBP1 in thyroid cancer tissues and cells, especially in ATC. In vitro and in vivo study found that SBP1 promotes the cell growth and dedifferentiation of thyroid cancer. SBP1 directly interacts with TXN, thus downregulating NIS expression and promotes tumorigenesis.

In the literature, the relationship between SBP1 and tumor growth is controversial. Levels of SBP1 have often been found to be decreased in diversity of cancers types and lower levels are often associated with poor clinical outcome (Kim et al. 2006; Kipp 2020; Li et al. 2008; Xia et al. 2011; Zhang et al. 2011). Exception was found in ovarian cancer where the expression of SBP1 was reduced in invasive ovarian cancer, while higher levels of SBP1 were associated with poor survival (Huang et al. 2006). In our studies to explore the role of SBP1 in thyroid cancer, we have found that SBP1 is highly expressed in thyroid cancer tissues and cells, especially in ATC. More interesting was the finding that knockdown of SBP1 inhibits tumor growth and progression of thyroid cancer, and promotes cell differentiation of BHT101 cells in a TXN/NIS dependent manner. Quantitative proteomic analysis reveals SBP1-mediated cancer inhibition is through altering lipid/glucose metabolic signaling pathways (Ying et al. 2015a, b). Loss of SBP-1 induces increased CXCR4 expression and enhanced invasive and poor prognosis of hepatocellular carcinoma (Gao et al. 2018). What is more, SBP1 negatively regulates oxidative phosphorylation in the healthy prostate cells by the production of H_2_O_2_ and H_2_S and consequential activation of AMPK (Elhodaky et al. 2020). These contrasting roles of SBP1 in different cancer type indicate distinct roles of SBP1 in the development of cancers of different origins. The distribution of SBP1 between cellular compartments may be relevant to cancer etiology. It is reported that a lower nuclear to cytoplasm ratio of SBP1 were associated with a higher tumor grade in prostate cancer and the level of SBP1 might be useful in distinguishing indolent from aggressive disease (Ansong et al. 2015). Here we found a strong nuclear staining of SBP1 in thyroid normal tissues and strong plasma staining of SBP1 in thyroid cancer tissues, suggesting that the translocation of nucleus to cytoplasm is related to the degree of malignancy of thyroid carcinoma. Considering that selenium and cancer is complicated by the existence of a diverse array of organic and inorganic selenium compounds, each with distinct biological properties, and this must be taken into consideration in the interpretation of both observational and experimental human studies (Vinceti et al. 2017).

It is reported that NIS expression is regulated by TXN/Ape1 through a TSH/Se-dependent mechanism (Leoni et al. 2016). TXN is a small multi-functional redox protein that undergoes NADPH-dependent reduction by thioredoxin reductase and in turn reduces oxidized cysteine groups of proteins. Studies found that TXN is overexpressed in most cancers, which can be responsible for drug resistance in tumorigenesis (Jia et al. 2019; Mohammadi et al. 2019). In the cytoplasm and nuclei of thyroid cancer cells, the level of TXN was increased when compared to normal thyroid tissue, and secreted TXN can enhance the sensitivity of the cells (Lincoln et al. 2010), which is in accordance with our finding that TXN is highly expressed in thyroid cancer patients and positively correlated with the thyroid cancer stage. Co-IP assay demonstrated that SBP1 direct interacts with TXN. Whether this interaction between SBP1 and TXN requires the selenocysteine in TXN or the selenium moiety of SBP1 is not known. Previous study showed that cysteine 57 of SBP1 was the likely selenium binding site because it is the only freely-accessible cysteine exposed to solvent and surrounded by hydrophobic residues (Raucci et al. 2011). Lately, studies have indicated that cysteine 57 is the most likely candidate amino acid for selenium binding. The half-life of SBP1, as well as the cellular response to selenite cytotoxicity, was altered by changing the cysteine 57 to a glycine, indicating that cysteine 57 is a critical determinant of SBP1 function and may play a significant role in mitochondrial function (Ying et al. 2015a, b). The exchange of cysteine 57 with glycine may probably a consequence of increase of TXN in BHT101 cells.

## Conclusions

In summary, we demonstrated that SBP1 promotes cell proliferation, tube formation, and inhibits cellular differentiation in differentiated thyroid cancer cell line *in vitro.* SBP1 knockdown, however, inhibits the growth and tumorigenesis of thyroid cancer in vitro and in vivo. Mechanically, SBP1 interacts with and promotes TXN, which reduces NIS expression in thyroid cancer. SBP1 knockdown represents a therapeutic alternative strategy for thyroid cancer.

### Electronic supplementary material

Below is the link to the electronic supplementary material.

Supplementary Material 1

Supplementary Material 2

## Data Availability

The datasets used and/or analyzed during the current study are available from the corresponding author on reasonable request.

## Abbreviations

ATC: Anaplastic thyroid cancer
DMEM: Dulbecco’s Modified Eagle’s medium
DTC: Metastatic differentiated thyroid cancer
FBS: Fetal bovine serum
FTC: Follicular thyroid carcinoma
IF: Immunofluorescence
IHC: Immunohistochemistry
MTO: Methanethiol oxidase
PDTC: Poorly differentiated thyroid cancer
PTC: Papillary thyroid carcinoma
RCECs: Retinal capillary endothelial cells
SBP1: Selenium-binding protein 1
siRNA: Small interfering RNA
TSH-R: Thyroid stimulating hormone receptor
TTF-1: Thyroid transcription factor-1
TTF-2: Thyroid transcription factor-1
TXN: Thioredoxin
VDU1: Von Hippel-Lindau protein (pVHL)-interacting deubiquitinating enzyme 1
