# Supplementary material for: SBP1 promotes tumorigenesis of thyroid cancer through TXN/NIS pathway
Source: Mol Med. 2023 Sep 8;29:121. doi: 10.1186/s10020-023-00700-y (PMC10492376; doi:10.1186/s10020-023-00700-y)
Supplement: Supplementary file 1 — Supplementary Material 1 [file 10020_2023_700_MOESM1_ESM.docx]

Dear editor,

We would like to submit the enclosed manuscript entitled “**SBP1 promotes tumorigenesis of thyroid cancer through TXN/NIS pathway**” to your honored journal **Molecular Medicine.** All authors have directly accessed and verified the data reported in the manuscript. The manuscript has not been previously submitted to or accepted by any other journal, that is has been approved by all authors, and the authors declare that they have no conflict of interest.

As the tissue with the highest selenium content in the body, the occurrence and development of thyroid cancer are closely related to selenium and selenoproteins. Selenium-binding protein (SBP) 1 has been repeatedly implicated in several cancers, but its role and molecular mechanisms in thyroid cancer remains largely undefined. The present study demonstrated that SBP1 was highly expressed in human thyroid cancer tissues and cells, especially in anaplastic thyroid cancer (ATC). Knockdown of SBP1 repress cell growth of BHT101, and the culture supernatant of SBP1-knockdown BHT101 repressed the tube formation of human retinal microvascular endothelial cells. However, SBP1 overexpression has the opposite effects as it increased cell growth and tube formation. What is more, overexpression of SBP1 inhibited cellular differentiation of differentiated thyroid cancer cell line FTC-133, as indicating by decreased expression of thyroid stimulating hormone receptors, thyroglobulin and sodium/iodide symporter (NIS). Knockdown of SBP1, however, promotes the differentiation of BHT101 cells, an anaplastic thyroid cancer cell line. Notably, SBP1 up-regulated the expression of thioredoxin (TXN), a negative regulator of NIS. Co-IP assay implied a direct interaction of SBP1 with TXN. Overexpression of TXN reversed the effect of SBP1 knockdown on BHT101 cell differentiation. In vivo study found that knockdown of SBP1 inhibits tumor growth and progression of thyroid cancer, as well as induces thyroid stimulating hormone receptors, thyroglobulin and NIS expressions. Taken together, all these data suggested that SBP1 promoted tumorigenesis and dedifferentiation of thyroid cancer through positively regulating TXN.

We hope this paper is suitable for your honored journal, and appreciate your consideration for our manuscript.

Best regards,

Xuejun Sun,

Department of General Surgery, The First Affiliated Hospital of Xi’an Jiaotong University, No. 277 West Yanta Road, Xi’an, 710061, Shaanxi, China.

E-mail: sunxy@mail.xjtu.edu.cn

Lieting Ma,

Department of Laboratory Medicine, The First Affiliated Hospital of Xi’an Jiaotong University, No. 277 West Yanta Road, Xi'an, 710061, Shaanxi, China.

E-mail: [mltmed@mail.xjtu.edu.cn](mailto:mltmed@mail.xjtu.edu.cn)
