# Supplementary material for: SBP1 promotes tumorigenesis of thyroid cancer through TXN/NIS pathway
Source: Mol Med. 2023 Sep 8;29:121. doi: 10.1186/s10020-023-00700-y (PMC10492376; doi:10.1186/s10020-023-00700-y)
Supplement: Supplementary file 2 — Supplementary Material 2 [file 10020_2023_700_MOESM2_ESM.docx]

Dear editor:

We quite appreciate your favorite consideration and the reviewers’ insightful comments to our manuscript “SBP1 promotes tumorigenesis of thyroid cancer through TXN/NIS pathway” (Ms. No. MOME-D-22-00856). Based on the current comments and suggestions, we have made carefully addresses, point-by-point response. All changes made to the text are in red color. The details are indicated in the marked version of the revised manuscript in the item “Revised manuscript 2”. The detailed corrections are listed below point by point:

Reviewer #2:

1. There are still several terms to define (write out term and but abbreviation in parentheses after) including CCK-8 and Co-IP.

Response: As what you have recommended, we have defined the CCK-8 and Co-IP in the revised manuscript. **(See Abstract section, See Page 11, line 9, and See Page 13, line 3)**

2. The authors state they independently verified one cell line, but didn't not address verification of the other (FTC line) as requested by 1st reviewer.

Response: Thanks for your question. In order to test the role of SBP1 on cell growth, transfection experiments were performed. Overexpression was tested in FTC-133 cell line and knockdown experiments were performed in BHT101 cell line. The 1st reviewer wanted us to explain why we use different cell lines. As answered previously, compared to normal thyroid cells, SBP1 is highly expressed in the FTC-133, B-CPAP, BHT-101 and ACT-1 cells, with the highest expression in BHT-101 cells **(Figure 1D)**. In addition, FTC-133 cell is a differentiated thyroid cancer cell line which is commonly used, while BHT-101 cell is a thyroid anaplastic carcinoma cell line derived from an undifferentiated primary carcinoma of the thyroid. To evaluate the effect of SBP1 in both differentiated and undifferentiated thyroid cancer cell line, overexpression of SBP1 was tested in FTC-133 cell and knockdown experiments were performed in BHT101 cell. The choice criteria are addressed in the Result section **(See Page 15, lines 13-14; and Page 15, lines 18-20)**.

3. For GEPIA analysis, the details of the samples/data entered and scientific principals and comparison approach/stats inherent in the software should be described, not the step by step instructions to compare results in the program.

Response: We appreciated your suggestion very much. We have added the details of the samples/data entered and scientific principals and comparison approach/stats inherent in the software. Based on the Cancer Genome Atlas (TCGA) and the Genotype-Tissue Expression (GTEx) projects, GEPIA2 is an interactive web for processing high-throughput RNA sequencing data of tumorous and normal samples. The present analysis included 512 thyroid carcinoma samples and 337 normal thyroid tissue samples. The “Box Plot” in the expression DIY module was used for analyzing the expression level of TXN in thyroid carcinoma samples and normal thyroid tissue samples. *P* value was set to 0.01, and the cut-off value of log_2_FC was set to 1. The “Stage Plot” in the expression DIY module was used to generate TXN expression violin plots based on the pathological stage of thyroid cancer patients. Major stage was used for plotting and the log_2_(TPM + 1) was used for Log scale. We have cited the reference on GEPIA in the revised manuscript ^[1]^. Besides, we have deleted the step by step instructions as what you have suggested. **(See Page 12, lines 6- 16).**

[1] Tang Z, Li C, Kang B, Gao G, Li C, Zhang Z. GEPIA: a web server for cancer and normal gene expression profiling and interactive analyses. Nucleic acids research. 2017;45(W1):W98-w102.

4. Please address how and why the control group went from 6 individuals to 12 individuals between the last draft and this draft of manuscript. What was the projected size of control group initially calculated to provide the desired statistical results?

Response: Thanks for your question. In fact, we have been collecting clinical samples for further research. In the last draft, we did not calculate the projected size of control group because we just detected the expressions of SBP1 and TXN in thyroid cancer tissues and adjacent normal tissues. Because the 1st reviewer requested us to investigate the correlation between SBP1, TXN and NIS expression in tumoral tissue, we calculated the sample size using G-power software. We have chosen <A priori: Compute required sample size – given α, power, and effect size> as type of power analysis and <t-tests (correlation: point biserial model)> as type of test. Enter α = 0.05, power = 0.90, and effect size = 0.7406 (or effect size = 0.8007) **(See Figures below)**. The effect size was calculated in the preliminary experiments based on the previous 6 clinical samples, and it was 0.7406 when evaluating the correlation between SBP1 and NIS expression, and it was 0.8007 while analyzing the correlation between SBP1 and TXN expression. The required sample size amounts to N = 11. As a result, we use 12 individuals in this draft of manuscript.

5. Finally, the conclusions section is brief and does not provide enough detail on the findings of the authors, please expand.

Response: We appreciated your suggestion very much. The expanded conclusions are as follows: In summary, we demonstrated that SBP1 promotes cell proliferation, tube formation, and inhibits cellular differentiation in differentiated thyroid cancer cell line *in vitro.* SBP1 knockdown, however, inhibits the growth and tumorigenesis of thyroid cancer *in vitro* and *in vivo*. Mechanically, SBP1 interacts with and promotes TXN, which reduces NIS expression in thyroid cancer. SBP1 knockdown represents a therapeutic alternative strategy for thyroid cancer. **(See Page 21, lines 8-15)**

We appreciate for Editors and Reviewers’ warm work earnestly, and hope the correction will meet with approval. Once again, thank you very much for your work concerning my paper.

Best regards,

Xuejun Sun,

Department of General Surgery, The First Affiliated Hospital of Xi’an Jiaotong University, No. 277 West Yanta Road, Xi’an, 710061, Shaanxi, China.

E-mail: sunxy@mail.xjtu.edu.cn

Lieting Ma,

Department of Laboratory Medicine, The First Affiliated Hospital of Xi’an Jiaotong University, No. 277 West Yanta Road, Xi'an, 710061, Shaanxi, China.

E-mail: mltmed@mail.xjtu.edu.cn
